# A Complicated Case of *Serratia marcescens* Infective Endocarditis in the Era of the Current Opioid Epidemic

**DOI:** 10.1155/2018/5903589

**Published:** 2018-11-19

**Authors:** Ho-Man Yeung, Brijaé Chavarria, Dariush Shahsavari

**Affiliations:** Department of Medicine, Lewis Katz School of Medicine at Temple University, Philadelphia, USA

## Abstract

While bacteremia due to *Serratia marcescens* is not uncommon, it rarely causes infective endocarditis. We report an isolated case of a 53-year-old male with history of intravenous drug abuse who presented with multiple acute pain symptoms and was found to have *S. marcescens* bacteremia with septic emboli in his spleen, brain, and testes, secondary to a large aortic vegetation, as well as aortic infective endocarditis with severe aortic regurgitation requiring aortic valve replacement. His course of disease was further complicated by epidural and psoas abscesses and a necrotic testicle requiring orchiectomy due to his ongoing intravenous drug abuse. This case is an atypical presentation of *S. marcescens* infection, as he had no overt signs of infection such as fever or significant leukocytosis that are typical of bacteremia, and it also highlights the severity and complicated nature of *S. marcescens*-infective endocarditis.

## 1. Background

According to the United States Centers for Disease Control and Prevention, opioid-related drug-overdose death continues to rise in the U.S., reaching an epidemic level. Intravenous drug users (IVDU) are not only at risk to blood-borne infections such as HIV and hepatitis, but also endocarditis. In such hosts, the most common organisms to cause endocarditis are Gram-positive cocci. However, in the current epidemic, clinicians need to be aware of atypical organisms as well.

*Serratia marcescens* (*S. marcescens*) is a facultative anaerobic, oxidase-negative, nonlactose-fermenting Gram-negative bacillus of the Enterobacteriaceae family. *S. marcescens* is commonly found in various environments including water, soil, plants, animals, and insects, but it is not a part of the human commensal flora. It is the main pathogen in the *Serratia* genus to cause human infections, most often associated with hospital settings and medical exposure. It is known to cause urinary tract infection, pneumonia, wound infections, skin and soft tissue infections, surgical site infection, as well as bloodstream infection [1]. Risk factors associated with such nosocomial infections include immunosuppression, previous antimicrobial agents, and indwelling catheterization. Despite being known to cause a wide spectrum of human infections, *S. marcescens* rarely causes endocarditis. Infective endocarditis by *S. marcescens* was first described in the medical literature as a case series of 19 patients observed in the San Francisco Bay Area, 17 of which were intravenous drug users [2]. Since then, only a handful of cases had been reported [3–9]. According to one study based on observations from the International Collaboration on Infective Endocarditis Prospective Cohort Study (ICE-PCS) database, only 0.14% of endocarditis cases are caused by the *Serratia* species [10].

## 2. Case Presentation

A 53-year-old Hispanic man with past medical history significant for coronary artery disease, habitual intravenous heroin abuse, chronic untreated hepatitis C without cirrhosis, bipolar disorder, tobacco abuse of 80 pack-year, and degenerative disc disease presented from home to our hospital with chief complaint of left scrotal pain, diffuse abdominal pain, back pain, and chest pain. Four days prior to presentation, he was seen in the emergency department for chronic back pain and was discharged with naproxen and instruction to follow up with his primary care physician. Since then, he developed acute onset of sharp constant pain of his left testicle and his abdomen. He admitted to using ten bags of heroin intravenously daily. He underwent an incision and drainage one month prior for a skin abscess. He reported no new sexual partner and is only sexually active with his current girlfriend. Family history was significant for heart disease in both his parents and maternal grandparents. His vital signs on admission were within the normal range. Physical exam revealed a man, with cachexia and temporal muscle wasting, in moderate distress from pain. He had jaundice with icteric sclera. His lungs were clear to auscultation. His heart sounds had regular rate and rhythm without any audible murmur. He had a soft abdomen that was mildly distended and tender in all four quadrants. His genitourinary exam was significant for bilateral scrotal erythema and swelling, which was worse on the left and was tender to light touch. He had diffuse tenderness to his back including paraspinal muscles, but no tenderness to the spinal processes. He was alert and oriented to person, time, and place and could answer questions appropriately. He had no facial asymmetry or deviated tongue, and he displayed normal proximal and distal strength. Laboratory findings were significant for WBC of 12.1 K/mm^3^ with a neutrophil predominance of 90%, microcytic anemia with hemoglobin 7.7 g/dL and MCV 75.6 fL, and thrombocytopenia of 47 K/mm^3^. His direct bilirubin level was elevated (3.0 mg/dL), as were his alkaline phosphatase (282 U/L), AST (87 U/L), ALT (33 U/L), and LDH (393 U/L). His albumin was 1.7 g/dL and lactic acid was normal at 1.2 mmol/L. His HIV-screening test was negative, as were his gonorrhea and chlamydia molecular amplification tests. He was subsequently admitted to internal medicine service/floor for further management.

**Figure 1:**
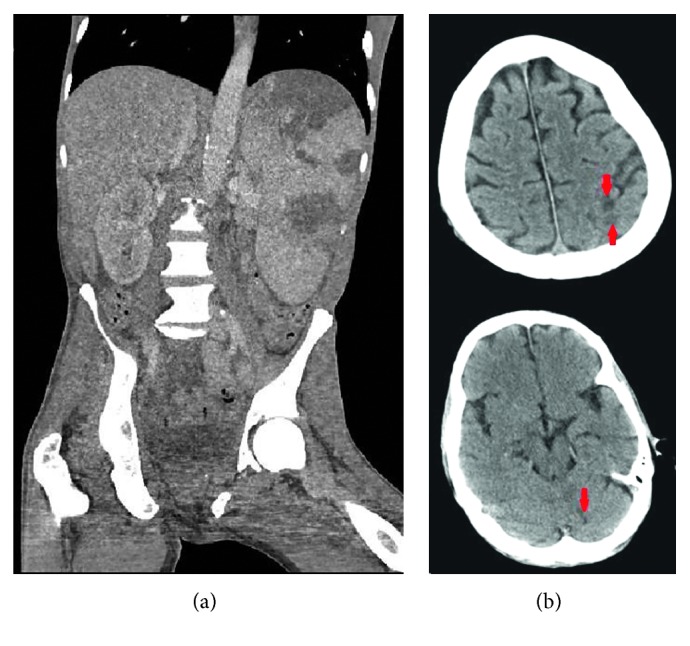
(a) Computed Tomography (CT) of the abdomen and the pelvis without contrast showing large wedge-shaped splenic infarcts. (b) CT of head without contrast displaying hypodensities in the left parietal lobe and left cerebellum due to septic emboli, shown in red arrows.

His initial work up included a urinalysis that was significant for 10–20 RBC per HPF, 5–10 WBC per HPF, and positive urine nitrite; an EKG that showed normal sinus rhythm; and a chest X-ray which was negative for cardiomegaly, pleural effusions, or lung consolidations. A computed tomography (CT) of his head did not show any intracranial abnormalities. A CT of his abdomen and pelvis without contrast was performed which revealed hepatosplenomegaly with multiple large wedge-shaped areas of hypoperfusion in the spleen, concerning splenic infarctions (Figure 1(a)). The scrotal ultrasound showed no torsions, but revealed left epididymo-orchitis, large complex left scrotal hydrocele, and bilateral testicular microlithiasis. A limited bedside transthoracic echocardiogram by the emergency department physicians revealed a small pericardial effusion with no observable valvular vegetations. Blood and urine cultures were collected, and he was initially started on the broad-spectrum antibiotics of vancomycin, piperacillin-tazobactam, and azithromycin considering his risk factors and clinical presentation.

Overnight, he remained afebrile, but his leukocytosis worsened to 14.6 K/mm^3^, and he developed dyspnea and tachypnea with 24 breaths per minute. He became disoriented and was not answering questions appropriately. On exam, new findings of splinter hemorrhages, toe infarctions, and subconjunctival hemorrhaging were noted. Urine culture grew *Serratia marcescens* 50,000–100,000 CFU. Blood cultures also later grew the same organism, susceptible to all tested antibiotics including amikacin, aztreonam, cefepime, ceftriaxone, ciprofloxacin, gentamicin, ertapenem, piperacillin/tazobactam, and trimethoprim/sulfamethoxazole. On hospital day 2, a formal transthoracic echocardiogram (TTE) revealed an ejection fraction of 60% (unchanged from three years prior) with a large echodensity on the aortic valve consistent with vegetation. Mild aortic regurgitation, mild mitral regurgitation, and mild tricuspid regurgitation were also noted. Antibiotics were subsequently changed toertapenem 1 g daily and ciprofloxacin 750 mg twice daily in consultation with infectious disease.

**Figure 2:**
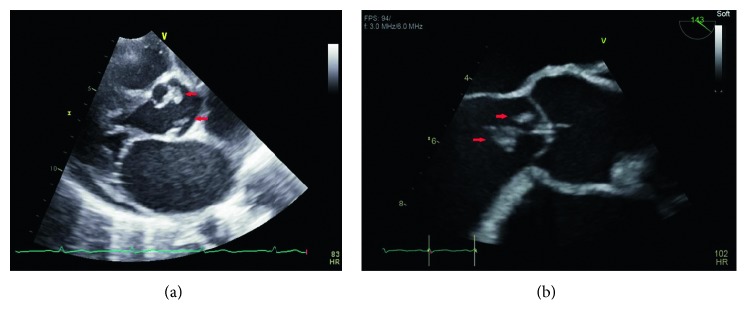
Large aortic valve vegetations on echocardiogram, shown in red arrows.

The patient continued to deteriorate the following day. He became more disoriented and agitated. He remained afebrile, but his tachypnea worsened to 30 breaths per minute, and new rales were now audible on lung auscultation. An arterial blood gas (pH 7.51; pCO_2_ 21 mmHg; pO_2_ 70 mmHg; HCO_3_ 16 mmol/L; O_2_ saturation 93%) showed a respiratory alkalosis. A repeat chest X-ray showed new interstitial pulmonary edema and vascular congestion. A CT of the head without contrast was performed due to his altered mental status, which showed two areas concerning embolic infarcts in the left parietal lobe and the left cerebellum (Figure 1(b)), compared to the one obtained on admission. On hospital day 4, an urgent transesophageal echocardiogram (TEE) was performed which again demonstrated the large aortic valve vegetations (Figure 2), but it now demonstrated acutely deteriorating moderate to severe aortic regurgitation, which was different from the TTE findings a few days prior that only showed mild-moderate aortic insufficiency. In addition, it also revealed a mildly dilated left ventricle. The aortic valve leaflet had prolapsed motion. Due to the presence of large aortic valve vegetations, multiple embolic phenomena, and a progressively worsening of his clinical condition, decision was made to perform an urgent aortic valve replacement.

Postoperatively, he was managed in the surgical intensive care unit, where he developed a moderate-sized left pneumothorax requiring a chest tube placement. Slowly, his mental and clinical status began to improve. By hospital day 19, the patient was no longer agitated or disoriented. He displayed good appetite, and his chest tube was removed. His leukocytosis significantly improved to 8.0 K/mm^3^, and his repeat blood cultures did not grow any organisms for 5 days. Throughout his hospital course, he remained afebrile and did not receive any antipyretics. The lack of fever was concerning for an immunocompromised state, but he tested negative for HIV. He was ultimately discharged to a skilled nursing facility to complete his antibiotics, ertapenem 1 g daily with ciprofloxacin 750 mg twice daily, for a total course of 6 weeks, and to continue physical therapy.

## 3. Follow-Up

One week after discharge, the patient returned to the hospital due to persistent fever and lower back pain. Given new fever and lower back pain, an urgent spine MRI was obtained and showed epidural abscess and multiple bilateral psoas muscle abscesses, presumed to be related to the prior *Serratia marcescens* infection. Surgical decompression of L2-L3 intervertebral space and discectomy as well as lumbar epidural abscess irrigation and drainage were performed. However, cultures did not grow any organisms as the patient was on antibiotics. The same antibiotic regimen was extended for two additional weeks to a total of 8 weeks, as he had breakthrough fevers. Repeat blood cultures showed no growth. Unfortunately, the patient eloped from the hospital before a repeat TTE was performed, and he did not complete his antibiotic course. He returned one week after with persistent back pain and testicular pain. He was found to have a necrotic left testicle and recommended an outpatient left orchiectomy, but he was absent to his scheduled time and never rescheduled. He was offered inpatient drug rehabilitation after completing his antibiotics, but he declined and elected outpatient rehabilitation. He follows up with our clinic for buprenorphine-naloxone therapy and attends narcotic anonymous; however, he continues to use IV heroin intermittently to treat his pain. At the time that this manuscript was written, he was admitted for septic shock and was found to have pseudomonal prosthetic valve endocarditis and an aortic root abscess with a VSD and an aorto-right atrial fistula secondary to the abscess. He underwent extensive cardiac surgery including an aortic valve replacement with pulmonary autograft, VSD closure, aorto-right atrial fistula closure, and left-atrial appendage ligation. He subsequently developed third-degree heart block requiring pacemaker placement. His current antibiotic regimen is cefepime and tobramycin for pseudomonal endocarditis.

## 4. Discussion

Infective endocarditis with *Serratia marcescens* is rare. While historically found to be associated with intravenous drug use, recent reports and studies have identified healthcare exposure as the source of infections [11–14]. However, on presentation, our patient did not have any implanted device or prior hospitalization to the best of our knowledge that would put him at risk for Serratia infections; therefore, IV drug abuse might be the cause of his disease. When he first presented to the emergency department, he did not have clear signs of infection such as fever or leukocytosis as one would expect from bacteremia. The infectious investigation was only initiated after findings of significant splenic infarctions on the CT abdomen that were highly suspicious for embolic phenomenon. Upon presentation, the infection had already disseminated, and the source of his bacteremia was unclear. Although primary bacteremia could have disseminated into his genitourinary tract, it is also possible that he had a common Serratia urinary tract infection as his primary source spreading into his bloodstream. Intravenous drug users are known to have higher rates of right-sided endocarditis. In contrast, *Serratia* seems to have a trend for left-sided valvular involvement, despite its association with IVDU, of which the mechanism remains unexplained to date [1]. Similar to previous cases, our patient was found to have left-sided valvular involvement that required surgical intervention. Gram-negative rods do not typically cause endocarditis in IVDU, but interestingly this patient suffered from two separate courses of Gram-negative rod-related endocarditis, *S. marcescens* native-valve infective endocarditis followed by pseudomonal prosthetic-valve endocarditis. It is unclear why this patient is particularly susceptible to Gram-negative endocarditis compared to the more typical Gram-positive organisms in IV drug users.

The sonographic findings throughout the case were interesting. Echocardiograms, in general, are operator-dependent and experience-dependent. The initial bedside echocardiogram was performed by an emergency medicine resident, and the images were reviewed by an emergency medicine clinician, compared to a formal echocardiogram, in which the images were obtained by an ultrasonographer and reviewed by a seasoned cardiologist. In fact, the bedside study was falsely reassuring and delayed the formal TTE or TEE study. If the bedside echocardiogram was able to detect the aortic valve vegetation, the patient would have been evaluated directly by TEE rather than TTE followed by TEE.

This case is especially challenging due to the patient's IV drug abuse behavior, compromising his treatment course and putting him at greater risk for repeated exposure to pathogens, which could threaten his prosthetic valve. Due to the production of AmpC beta-lactamase, *Serratia marcescens* is generally resistant to penicillins and first- and second-generation cephalosporins, making it imperative to consider susceptibilities when determining appropriate antibiotic therapy [15]. Additionally, acquisition of extended spectrum beta-lactamases and carbapenemases has also been documented [16, 17]. Guidelines for antimicrobial therapy for Serratia endocarditis are not well defined, although the 2015 Infectious Diseases Society of America (IDSA) infective endocarditis guideline suggests combination therapy with a *β*-lactam and an aminoglycoside or fluoroquinolone for 6 weeks [18]. In summary, *Serratia marcescens* infective endocarditis is a very rare infection with no definitive treatment guidelines. In the era of national opioid epidemics and increased risk of blood-borne infections, it is imperative to recognize these less-frequent organisms as the cause of infective endocarditis and initiate the appropriate treatment promptly.
